# Identification of candidate serum biomarkers for severe septic shock-associated kidney injury via microarray

**DOI:** 10.1186/cc10554

**Published:** 2011-11-18

**Authors:** Rajit K Basu, Stephen W Standage, Natalie Z Cvijanovich, Geoffrey L Allen, Neal J Thomas, Robert J Freishtat, Nick Anas, Keith Meyer, Paul A Checchia, Richard Lin, Thomas P Shanley, Michael T Bigham, Derek S Wheeler, Prasad Devarajan, Stuart L Goldstein, Hector R Wong

**Affiliations:** 1 Cincinnati Children's Hospital Medical Center and Cincinnati Children's Research Foundation, Department of Pediatrics, University of Cincinnati College of Medicine, 3333 Burnet Avenue, Cincinnati, OH 45223, USA; 2 Children's Hospital and Research Center Oakland, 747 52nd Street, Oakland, CA 94609, USA; 3 Children's Mercy Hospital, 2401 Gillham Road, Kansas City, MI 64108, USA; 4 Penn State Children's Hospital, 500 University Drive, Hershey, PA 17033, USA; 5 Children's National Medical Center, 111 Michigan Avenue, NW, Washington, District of Columbia 20010, USA; 6 Children's Hospital of Orange County, 455 South Main Street, Orange, CA 92868, USA; 7 Miami Children's Hospital, 3100 SW 62nd Avenue, Miami, FL 33155, USA; 8 Texas Children's Hospital, 6621 Fannin Street, Houston, TX 77030, USA; 9 The Children's Hospital of Philadelphia, 34th Street and Civic Center, Philadelphia, PA 19104, USA; 10 CS Mott Children's Hospital at the University of Michigan, 1500 East Medical Center Drive, Ann Arbor, MI 48109, USA; 11 Akron Children's Hospital, 1 Perkins Square, Akron, OH 44308, USA

## Abstract

**Introduction:**

Septic-shock-associated acute kidney injury (SSAKI) carries high morbidity in the pediatric population. Effective treatment strategies are lacking, in part due to poor detection and prediction. There is a need to identify novel candidate biomarkers of SSAKI. The objective of our study was to determine whether microarray data from children with septic shock could be used to derive a panel of candidate biomarkers for predicting SSAKI.

**Methods:**

A retrospective cohort study compared microarray data representing the first 24 hours of admission for 179 children with septic shock with those of 53 age-matched normal controls. SSAKI was defined as a >200% increase of baseline serum creatinine, persistent to 7 days after admission.

**Results:**

Patients with SSAKI (*n *= 31) and patients without SSAKI (*n *= 148) were clinically similar, but SSAKI carried a higher mortality (45% vs. 10%). Twenty-one unique gene probes were upregulated in SSAKI patients versus patients without SSAKI. Using leave-one-out cross-validation and class prediction modeling, these probes predicted SSAKI with a sensitivity of 98% (95% confidence interval (CI) = 81 to 100) and a specificity of 80% (95% CI = 72 to 86). Serum protein levels of two specific genes showed high sensitivity for predicting SSAKI: matrix metalloproteinase-8 (89%, 95% CI = 64 to 98) and elastase-2 (83%, 95% CI = 58 to 96). Both biomarkers carried a negative predictive value of 95%. When applied to a validation cohort, although both biomarkers carried low specificity (matrix metalloproteinase-8: 41%, 95% CI = 28 to 50; and elastase-2: 49%, 95% CI = 36 to 62), they carried high sensitivity (100%, 95% CI = 68 to 100 for both).

**Conclusions:**

Gene probes upregulated in critically ill pediatric patients with septic shock may allow for the identification of novel candidate serum biomarkers for SSAKI prediction.

## Introduction

Septic shock leads to significant morbidity and mortality in critically ill adult and pediatric patients [1,2]. Meanwhile, acute kidney injury (AKI) is also known to be independently associated with mortality and morbidity in critically ill patients. The treatment of sepsis costs the US population over $15 billion/year for adults and over $2 billion/year for children, while the costs for AKI approach $10 billion/year for adults alone [3,4]. Sepsis is the most common precipitant for AKI in both populations, and the development of kidney injury in the context of sepsis is a poor prognostic sign. Together the two disease processes carry up to 75% mortality [5-9].

Effective therapies for septic-shock-associated acute kidney injury (SSAKI) are lacking. Detection schemes for SSAKI have been and still are dependent on serum creatinine, a flawed real-time marker of AKI [10,11]. Diagnoses of SSAKI based upon changes in creatinine, therefore, are considerably varied and create heterogeneity between studies investigating AKI therapy. Biomarker research seeking to identify more robust markers of AKI has yielded promising results. Neutrophil gelatinase-associated lipocalin (NGAL), IL-18, and cystatin C have all demonstrated encouraging efficacy for predicting ischemic or nephrotoxic AKI and its severity [12-14]. Sepsis intrinsically induces increased expression of some AKI biomarkers (for example, NGAL), and studies of NGAL levels in patients with SSAKI often demonstrate high sensitivity with modest specificity [9,14-17].

Importantly, the pathophysiology of SSAKI may be unique from that of ischemic or nephrotoxic AKI [18]. Therapies aiming to restore renal perfusion in ischemic AKI [19-21] have not been demonstrated to be particularly effective and may be even less effective in SSAKI, a process that may not be secondary to impaired glomerular preload. Persistent SSAKI may fall into the class of fluid-unresponsive AKI [22]. Renal replacement therapy has been used as therapy for AKI and data exist demonstrating that initiation prior to accumulation of excessive fluid overload may improve outcomes [23,24]. The aggregate data, however, show that patients with SSAKI have consistently increased mortality, even with early renal replacement therapy initiation [25,26]. The modest efficacy of biomarkers at identifying SSAKI also underscores the notion that the pathophysiology of SSAKI is unique from other etiologies of AKI. There is a need to identify novel candidate biomarkers of SSAKI, which would expedite early treatment aimed at preventing the effects of this highly morbid complication of sepsis.

We have generated an extensive genome-wide expression database from children with septic shock by way of microarray technology and have now leveraged this database to identify candidate biomarkers for SSAKI [27-32]. Herein we report the identification of 21 unique gene probes upregulated in patients with SSAKI, within the first 24 hours of admission to the pediatric intensive care unit (PICU), and their ability to robustly predict SSAKI. Two readily measurable gene products from this list, matrix metalloproteinase-8 (MMP-8) and neutrophil elastase-2, show high sensitivity for SSAKI in a cohort of patients with septic shock.

## Materials and methods

### Patients and data collection

The study protocol was approved by the Institutional Review Boards of each participating institution (*n *= 11). Children ≤10 years of age admitted to the PICU and meeting pediatric-specific criteria for septic shock were eligible for enrollment [33]. Controls, used to normalize the microarray data across the patients with septic shock and to conduct the three-group analysis of variance in the first derivation analysis, were recruited from the ambulatory departments of participating institutions using published inclusion and exclusion criteria [28-32]. These controls were required to reliably compare data across different batches of samples. All patients and controls in the derivation cohort were previously reported in microarray-based studies addressing hypotheses entirely different from that of the current report [28-32]. All microarray data have been deposited in the NCBI Gene Expression Omnibus (GEO:GSE26440 and GEO:GSE26378). The patients in the validation cohort have not been previously reported in any manner.

After informed consent from legal guardians, blood samples were obtained within 24 hours of initial presentation to the PICU with septic shock. Clinical and laboratory data were collected daily while in the PICU, and were stored using a web-based database. Mortality was tracked for 28 days after enrollment.

SSAKI was defined as a >200% rise of serum creatinine relative to the median normal value for age [34,35], persistent to day 7 of PICU admission. For example, if a patient had a >200% rise of serum creatinine upon admission to the PICU but returned to <200% by day 7 (without renal replacement therapy), then that patient was classified as not having SSAKI. Although stringent, this definition allowed us to identify those patients with persistent fluid-unresponsive AKI. No patient in this study had known pre-existing chronic kidney disease. Patients who died before day 7 with a creatinine rise >200% from baseline were included.

### RNA extraction and microarray hybridization

Total RNA was isolated from whole blood using the PaxGene™ Blood RNA System (PreAnalytiX, Qiagen/Becton Dickson, Valencia, CA, USA). Microarray hybridization was performed as previously described using the Human Genome U133 Plus 2.0 GeneChip (Affymetrix, Santa Clara, CA, USA) [27-32].

### Serum protein biomarker measurements

Measurements of serum MMP-8 and elastase-2 protein levels were performed using a Luminex 200 multi-plex instrument (Luminex Corporation, Austin, TX, USA) and antibody-coated magnetic beads (Millipore, Billerica, MA, USA) following the manufacturer's specifications.

### Data analysis

Analyses were performed using one patient sample per chip. Image files were captured using an Affymetrix GeneChip Scanner 3000. CEL files were subsequently preprocessed using robust multiple-array average normalization and GeneSpring GX 7.3 software (Agilent Technologies, Palo Alto, CA, USA). All signal intensity-based data were used after robust multiple-array average normalization, which specifically suppresses all but significant variation among lower intensity probe sets [36]. All chips representing patient samples were then normalized to the respective median values of controls.

Differences in mRNA abundance between the study categories were measured using GeneSpring GX 7.3 software, as were the class prediction procedures. Receiver operating characteristic (ROC) curves and calculations of biomarker performance were performed using SPSS (IBM Corporation, Somers, NY, USA). Ordinal and continuous clinical variables not normally distributed were analyzed via analysis of variance on ranks. Dichotomous clinical variables were analyzed using a chi-square test (SigmaStat software; Systat Software, Inc., San Jose, CA, USA).

## Results

### Initial derivation of candidate biomarkers for SSAKI

Based on complete microarray data from 179 children with septic shock, we used a multistage approach to begin deriving a panel of candidate biomarkers to predict SSAKI. All of the microarray data in the derivation process represent the first 24 hours of admission to the PICU with septic shock. Table 1 provides the clinical characteristics of the derivation cohort, consisting of 148 patients without SSAKI and 31 patients with SSAKI. The patients with SSAKI had a higher Pediatric Risk of Mortality score and a higher mortality rate, compared with the patients without SSAKI. All other variables shown in Table 1 were not significantly different between the two groups.

**Table 1:** Clinical characteristics of the derivation cohort

Characteristic	No SSAKI (*n *= 148)	SSAKI (*n *= 31)
Age (years)	2.4 (1.0 to 6.0)	2.7 (0.8 to 8.4)
Males	87 (59)	22 (71)
Pediatric Risk of Mortality	14 (9 to 19)	22 (16 to 31)*
Deaths^a^	15 (10)	14 (45)*
With Gram-negative organism	43 (28)	4 (13)
With Gram-positive organism	37 (25)	10 (32)
With negative cultures^b^	57 (39)	14 (45)

Data presented as median (interquartile range) or number (%). Sepsis-shock-associated acute kidney injury (SSAKI) defined as a persistent >200% increase of serum creatinine at day 7 of hospitalization. ^a^Twenty-eight-day mortality. ^b^Refers to patients in which no pathogenic organism was isolated. The total percentage of organisms does not add up to 100% because patients with either a fungal or viral pathogen were not included. *P <0.05 versus patients without kidney injury.

In the first derivation stage we conducted a two-step statistical test to determine which gene probes on the array (>80,000 gene probes) were differentially regulated between patients with and without SSAKI. In step one we conducted a three-group analysis of variance using normal controls (*n *= 53), patients without SSAKI, and patients with SSAKI as the comparison groups, and corrections for multiple comparisons (Benjamini-Hochberg false discovery rate = 5%). This was followed by a *post hoc *test (Tukey) to isolate the gene probes differentially regulated between patients with and without SSAKI (100 gene probes, see Additional file 1).

**Figure 1:**
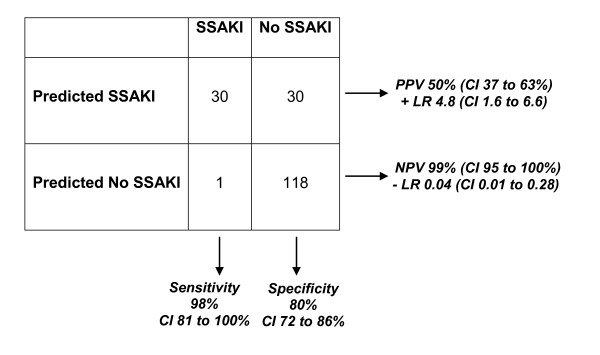
**Results of the leave-one-out cross-validation procedure involving 21 gene probes**. The procedure was based on a Support Vector Machines algorithm and was targeted at prediction of 'SSAKI' and 'no SSAKI' classes. Performance calculations provided as the percentage with 95% confidence intervals (CIs). LR, likelihood ratio; NPV, negative predictive value; PPV, positive predictive value; SSAKI, sepsis-shock-associated acute kidney injury.

The 100-gene probe list presented in Additional file 1 corresponds to 61 unique and well-annotated genes. Twenty-one of the gene probes were upregulated in the patients with SSAKI, relative to the patients without SSAKI (Table 2). These 21 gene probes were subsequently used in a leave-one-out cross-validation procedure (Support Vector Machines algorithm) to predict 'SSAKI' and 'no SSAKI' classes in the derivation cohort. The leave-one-out cross-validation procedure removes a single observation from the original sample as validation - and analyzes the remaining observations as comparators. The procedure is repeated for each observation in the sample so that each is used once as validation. Figure 1 provides the 2 × 2 contingency table demonstrating the results of the leave-one-out cross validation procedures, and the associated performance calculations. Figure 1 demonstrates that the expression patterns of these 21 upregulated gene probes can predict SSAKI with a high degree of sensitivity and modestly high specificity in the derivation cohort. In addition, the expression patterns of these 21 upregulated gene probes have a high negative predictive value for SSAKI in the derivation cohort. Accordingly these 21 gene probes represent potential candidate biomarkers for predicting SSAKI.

**Table 2:** Gene probes upregulated in patients with kidney injury that predict 'no SSAKI' and 'SSAKI' classes

Affymetrix ID	Fold change	Gene symbol	GenBank ID	Description
202411_at	2.088	IFI27	NM_005532	IFNα-inducible protein 27
207329_at	1.756	MMP8	NM_002424	Matrix metallopeptidase-8
212768_s_at	1.711	OLFM4	AL390736	Olfactomedin 4
219975_x_at	1.697	OLAH	NM_018324	Oleoyl-ACP hydrolase
206145_at	1.688	RHAG	NM_000324	Rh-associated glycoprotein
231688_at	1.666	MMP8	AW337833	Matrix metallopeptidase-8
233126_s_at	1.664	OLAH	AK001844	Oleoyl-ACP hydrolase
211820_x_at	1.612	GYPA	U00179	Glycophorin A
222945_x_at	1.612	OLAH	AI125696	Oleoyl-ACP hydrolase
220496_at	1.601	CLEC1B	NM_016509	C-type lectin domain family 1, member B
219478_at	1.601	WFDC1	NM_021197	WAP four-disulfide core domain 1
205110_s_at	1.551	FGF13	NM_004114	Fibroblast growth factor 13
206871_at	1.525	ELA2	NM_001972	Elastase-2, neutrophil
205612_at	1.522	MMRN1	NM_007351	Multimerin 1
219410_at	1.517	TMEM45A	NM_018004	Transmembrane protein 45A
207341_at	1.512	PRTN3	NM_002777	Proteinase 3
218542_at	1.509	CEP55	NM_018131	Centrosomal protein 55 kDa
211372_s_at	1.505	IL1R2	U64094	IL-1 receptor type II
207269_at	1.497	DEFA4	NM_001925	Defensin, α4, corticostatin
201292_at	1.492	TOP2A	AL561834	Topoisomerase (DNA) IIα 170 kDa
211821_x_at	1.486	GYPA	U00178	Glycophorin A

Twenty-one gene probes upregulated in patients with kidney injury and used for the leave-one-out cross-validation procedure to predict 'no SSAKI' and 'SSAKI' classes. SSAKI, sepsis-shock-associated acute kidney injury.

Matrix metalloproteinase-8 and elastase-2 serum levels as predictors of SSAKI

**Figure 2:**
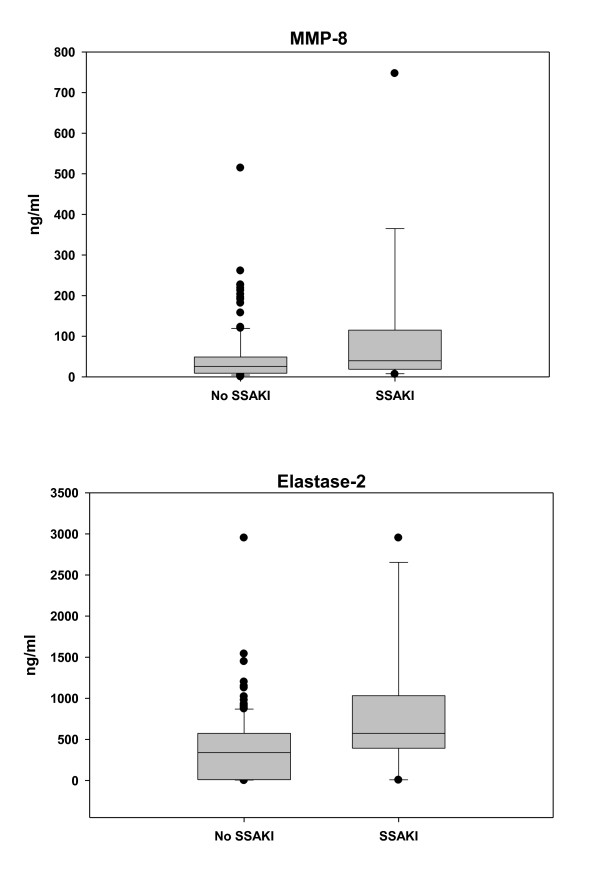
**Serum matrix metalloproteinase-8 and elastase-2 levels in patients with and without sepsis-shock-associated acute kidney injury**. Serum matrix metalloproteinase-8 (MMP-8) and elastase-2 levels are higher in patients with sepsis-shock-associated acute kidney injury (SSAKI) than patients without SSAKI. Patients with SSAKI (*n *= 132) were compared with patients without SSAKI (*n *= 18). Serum samples were obtained within 24 hours of admission to the pediatric ICU with septic shock and were drawn in parallel with RNA samples. Medians and interquartile ranges for the targets of interest for no SSAKI versus SSAKI: MMP-8, 26 (9 to 49) versus 40 (20 to 98), *P *= 0.029; and neutrophil elastase-2, 340 (9 to 571) and 574 (444 to 911), *P *= 0.01 (rank-sum test).

To further test the ability of the gene probes to predict SSAKI, we conducted initial measurements of selected gene probe products. Two gene probes corresponding to MMP-8 and one gene probe corresponding to elastase-2 were included in the 21 gene probes used in the class prediction modeling procedure described above. Since MMP-8 and elastase-2 protein levels can be readily measured in the serum compartment, we tested the ability of MMP-8 and elastase-2 serum protein levels to predict SSAKI in the derivation cohort. There were 150 parallel serum samples from the derivation cohort available for this analysis (132 patients without SSAKI and 18 patients with SSAKI). Figure 2 demonstrates that both MMP-8 and elastase-2 serum protein levels were higher in patients with SSAKI, compared with patients without kidney injury, thus corroborating the microarray data.

**Figure 3:**
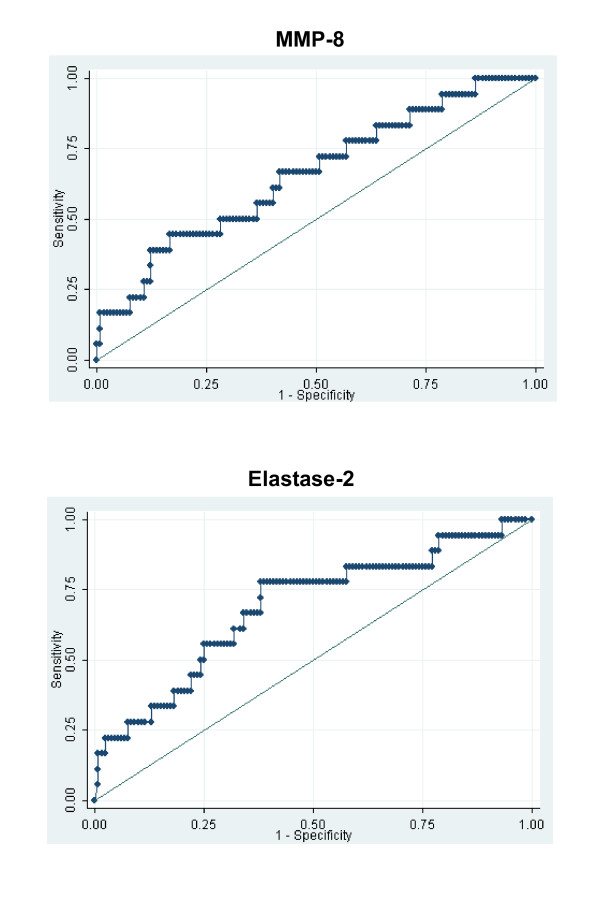
**Matrix metalloproteinase-8 and elastase-2 as candidate biomarkers for predicting sepsis-shock-associated acute kidney injury**. Receiver operating characteristic curves for matrix metalloproteinase-8 (MMP-8) and elastase-2 as candidate biomarkers for predicting sepsis-shock-associated acute kidney injury. Area under the curve = 0.659 (95% confidence interval = 0.520 to 0.798) and area under the curve = 0.688 (95% confidence interval = 0.549 to 0.826) for matrix MMP-8 and elastase-2, respectively.

We next constructed ROC curves to determine the ability of serum protein levels of MMP-8 and elastase-2, respectively, to predict SSAKI in the derivation cohort (Figure 3). From these ROC curves we empirically selected cut-off values of 11 ng/ml (MMP-8) and 235 ng/ml (elastase-2) with an *a priori *goal of achieving a high sensitivity at the expense of specificity. The performance characteristics of the respective cut-off values are shown in Table 3. For both MMP-8 and elastase-2, the respective cut-off values were able to predict SSAKI with a relatively high sensitivity. In addition, both cut-off values had a high negative predictive value for the development of SSAKI. When we incorporated both MMP-8 and elastase-2 into a multiple regression analysis, we were unable to substantially improve the ability to predict SSAKI compared with the ability of each candidate biomarker alone (data not shown).

**Table 3:** Individual performance calculations of serum protein levels for predicting SSAKI in the derivation cohort

Performance	MMP-8	Elastase-2
Sensitivity	89 (64 to 98)	83 (58 to 96)
Specificity	29 (21 to 37)	42 (34 to 51)
Positive predictive value	15 (9 to 23)	16 (10 to 26)
Negative predictive value	95 (82 to 99)	95 (85 to 99)

Data expressed as percentage (95% confidence intervals). MMP-8, matrix metalloproteinase-8; SSAKI, septic-shock-associated acute kidney injury.

### Validation of MMP-8 and elastase-2 performance

Having demonstrated the individual performances of MMP-8 and elastase-2 as candidate biomarkers for predicting SSAKI in the derivation cohort, we next applied the same respective cut-off values to a separate validation cohort of patients. The validation cohort consisted of 59 patients without SSAKI and 11 patients with SSAKI. The clinical characteristics of the validation cohort were not significantly different from those of the derivation cohort, except that in the validation cohort the patients with SSAKI had a higher proportion of males and a lower proportion of patients with negative cultures compared with the patients without SSAKI (Table 4).

**Table 4:** Clinical characteristics of the validation cohort

Characteristic	No SSAKI (*n *= 59)	SSAKI (*n *= 11)
Age (years)	2.1 (0.6 to 4.6)	1.4 (0.9 to 8.5)
Males	34 (58)	10 (91)*
Pediatric Risk of Mortality	10 (7 to 117)	25 (10 to 28)*
Deaths^a^	2 (3)	2 (19)
With Gram-negative organism	10 (17)	4 (36)
With Gram-positive organism	9 (15)	4 (36)
With negative cultures	40 (68)	3 (27)*

Data presented as median (interquartile range) or number (%). SSAKI (sepsis associated acute kidney injury) was defined as a persistent >200% increase of serum creatinine at day seven of hospitalization. ^a^Twenty-eight-day mortality. *P <0.05 versus patients without kidney injury.

Table 5 provides the respective performance calculations for the two candidate biomarkers in the validation cohort. Both candidate biomarkers were able to predict SSAKI with 100% sensitivity in the validation cohort. However, the markers carried limited positive predictive value. In the validation cohort, 35 patients had an MMP-8 level >11 ng/ml without sustained SSAKI. In these patients, eight (23%) met criteria for at least mild AKI (pediatric Risk, Injury, Failure, Loss, End-Stage kidney injury (RIFLE) Risk) for at least 1 day. Also in the validation cohort, 30 patients had an elastase-2 level >235 ng/ml without sustained SSAKI. OK Nine (30%) of these patients met at least pediatric RIFLE Risk criteria for at least 1 day. Both biomarkers had negative predictive values for SSAKI of 100%, an important finding that adds clinical utility for practitioners treating patients who carry risks for developing severe AKI. Collectively, our results indicate that admission microarray data can be used to identify novel candidate biomarkers for SSAKI.

**Table 5:** Individual performance calculations of serum protein levels for predicting SSAKI in the validation cohort

Performance	MMP-8	Elastase-2
Sensitivity	100 (68 to 100)	100 (68 to 100)
Specificity	41 (28 to 50)	49 (36 to 62)
Positive predictive value	24 (13 to 39)	27 (15 to 43)
Negative predictive value	100 (83 to 100)	100 (85 to 100)

Data expressed as percentage (95% confidence intervals). MMP-8, matrix metalloproteinase-8; SSAKI, septic-shock-associated acute kidney injury.

## Discussion

We have demonstrated that microarray data can be leveraged to identify gene expression patterns common in SSAKI. Although mRNA levels do not necessarily correlate with protein expression, analysis of MMP-8 and elastase-2 protein levels in the serum compartment indicates that our gene expression data can identify novel serum protein biomarkers for SSAKI.

Although sepsis is the most important predictor of AKI in critically ill patients [37,38], few clinical studies are dedicated to identifying the early phenotype of patients with SSAKI. Interrogation of several large databases of critically ill adult patients has yielded variables that may be predictive of SSAKI but are not consistently reliable (Table 6). The conclusions of these studies simply state that elevated proinflammatory markers portend adverse outcomes in acute kidney disease and death in patients with septic shock [39,40].

**Table 6:** Variables predictive of SSAKI in selected major adult trials

Study	Criteria	SSAKI (%)	Variable outcome
PROWESS [39]	Pre-infusion log IL-6, APACHE II score	127/547 (23.2)	Hazard ratio = 1.16 (95% CI = 1.07 to 1.26); *P *<0.001
			Hazard ratio = 1.23 (95% CI = 1.09 to 1.39); *P *<0.001
NORASEPT II [54]	Plasma IL-6	112/537 (20)	Not significant
	Plasma TNFα		Not significant
	Soluble TNFα receptor type I		Higher in SSAKI; *P *<0.0001
	Soluble TNFα receptor type II		Higher in SSAKI; *P *<0.0001
PICARD [40]	Plasma TNFα	34/97 (35)	Higher in patients with SSAKI (*P *= 0.001) than those without sepsis
	Plasma IL-6		Not significant
Hoste and colleagues [55]	APACHE II score	30/185 (16.2)	APACHE II score higher (*P *= 0.002)
	pH <7.35		OR for developing ARF = 6.25 (95% CI = 1.92 to 20.4); *P *= 0.002
	Serum creatinine >1 mg/dl		OR for developing ARF = 7.56 (95% CI = 2.16 to 26.5); *P *= 0.002
Martensson and colleagues [56]	Peak serum NGAL	18/45 (40)	Not significant in SSAKI vs. septic shock without AKI
	Peak urine NGAL		Higher in SSAKI than septic shock without AKI (*P *<0.05)
Shapiro and colleagues [43]	Emergency room plasma NGAL	24/661 (3.6)	NGAL >150: sensitivity = 96% and specificity = 51%
de Geus and colleagues [42]	Serum NGAL	171/632 (27)	OR of developing ARF = 1.7
	Urine NGAL		OR of developing ARF = 1.42
	Sepsis		OR of developing ARF = 9.15

AKI, acute kidney injury; APACHE, Acute Physiology and Chronic Health Evaluation; CI, confidence interval; NGAL, neutrophil gelatinase-associated lipocalin; NORASEPT, North American Study for the Safety and Efficacy of Murine Monoclonal Antibody to Tumor Necrosis Factor Alpha for the Treatment of Septic Shock; OR, odds ratio; PICARD, Program to Improve Care in Acute Renal Disease; PROWESS, Prospective Recombinant Activated Protein C Worldwide Evaluation in Severe Sepsis; SSAKI, sepsis-shock-associated acute kidney injury; TNF, tumor necrosis factor.

NGAL has received considerable attention as a potential biomarker for AKI. NGAL derivation and validation studies were primarily performed in ischemic or nephrotoxic AKI with great efficacy [13], while investigations of NGAL biomarker utility in SSAKI have demonstrated variable results. In the general ICU population, serum NGAL levels have not been reliably demonstrated to be specific for SSAKI (Table 6). We previously published that while day 1 serum NGAL levels were elevated, with acceptable sensitivity, in children who develop SSAKI, the specificity was quite poor (39%) [41]. A recent prospective cohort study of 632 adult ICU patients demonstrated that the likelihood ratio for AKI (RIFLE Failure) was 1.71 for NGAL, and that elevated NGAL alone conferred an ROC area under curve of 0.77 for RIFLE Failure but when included in a 'most efficient clinical model' NGAL improved the ROC area under curve for RIFLE Failure to 0.96 [42]. OK In this study, specificity was 50% for a plasma NGAL cut-off value of 168 ng/ml. Emergency room serum NGAL levels >150 ng/ml were highly sensitive for AKI within 72 hours (96%), but specificity was only 51% [43]. NGAL is known to be released by activated neutrophils and appears to be intrinsically elevated in sepsis, which may explain its limitation as a biomarker specific for SSAKI.

A variable that may complicate delineation of biomarkers for SSAKI is that the pathophysiology of SSAKI may be different from that of ischemic or nephrotoxic AKI. Although acute tubular necrosis has traditionally been considered the etiology of AKI in sepsis, no conclusive pathologic evidence has demonstrated that this is true [18]. Animal models of SSAKI demonstrate increased, rather than decreased, renal blood flow and a state of hyperdynamic AKI [44]. The mechanisms mediating disease progression are multifactorial, and AKI during sepsis may be a result of acute tubular *apoptosis *rather than acute tubular *necrosis *[18]. Nonhemodynamic-dependent mechanisms of injury during sepsis, inflammatory and immunologic, may trigger this apoptotic response. This pathophysiology has probably decreased the utility of traditional tests based on the functionality of proximal tubular filtration [45].

Our study demonstrates that microarray-derived gene expression data can provide a tool for discovery of candidate biomarkers for SSAKI. Outside the neonatal population, we are the first group to attempt to characterize biomarkers specific to SSAKI (and not all-cause AKI) in children. Our data represent the first 24 hours of presentation to medical care, a timeframe that can be considered a therapeutic window for acute intervention. It is important to note that our patients were restricted to having septic shock, an exclusion criterion not previously used in pediatric studies investigating AKI biomarkers. Additionally, our definition of SSAKI identifies patients with severe, persistent kidney injury up to day 7. These candidate biomarkers thus identify patients with resuscitation unresponsive AKI (that is, patients having high serum creatinine levels at admission who subsequently *did not *normalize with standard resuscitation). The expression patterns of the 21 upregulated gene probes demonstrate excellent sensitivity and good specificity for SSAKI (Figure 1). The negative predictive value of nearly 100% carries obvious import to the bedside practitioner, but should be tempered by the fact that the prevalence of AKI in our cohort was relatively low (31/179, 17%). Individual performance calculations of two of these gene products, MMP-8 and elastase-2, demonstrate reasonable sensitivity in the derivation cohort (Table 3), but even higher sensitivity in the validation cohort (Table 5). Additionally, in the validation cohort, the specificity and negative predictive values of MMP-8 and elastase-2 increased further. Although MMP-8 and elastase-2 will require further prospective validation studies for confirmation, the results are a relevant demonstration that our gene expression methodology can successfully be leveraged against the tremendous heterogeneity in patients with SSAKI.

The biological links between MMP-8, elastase-2, and SSAKI are unclear at the present time. MMP-8 is a collagen-cleaving endopeptidase, secreted by neutrophils, involved in extracellular matrix remodeling. MMP-8 is implicated in the pathogenesis of numerous rheumatologic diseases and has been demonstrated as a biomarker for disease processes ranging from odontogenic inflammation, to left ventricular remodeling after myocardial infarction, to preterm labor [46-48]. We have recently identified that MMP-8 may be important in the pathogenesis of septic shock [49], and Hu and colleagues demonstrated that the use of an MMP-8 inhibitor protects mice against endotoxic shock [50]. There are no previous reports of MMP-8 involvement in the progression of kidney injury. Neutrophil elastase (elastase-2) is a serine protease secreted by neutrophils in response to bacterial infection, is a marker of inflammation, and has been documented as a biomarker of neonatal sepsis [51]. Interestingly, inhibition of neutrophil elastase reduced progressive renal injury seen in experimental direct endotoxin-induced AKI [52].

Our study has several limitations. Since in many cases neither a baseline creatinine nor a height was available, the definition of SSAKI in our population was based on a referenced median-for-age creatinine [34,35]. Importantly, the initial pediatric RIFLE study also identified AKI in patients based on *assumed *estimated creatinine clearance when baseline creatinine levels were unavailable [53]. Second, some of the performance characteristics (negative and positive predictive values) are influenced by the relatively low prevalence of AKI in our cohort. This is probably secondary to the stringent *a priori *definition used for SSAKI. The intentional use of such a definition allowed us to identify the most severely affected patients with fluid-unresponsive AKI [22]. Third, the data for MMP-8 and elastase-2 continue to demonstrate the relatively low specificity that many candidate biomarkers carry for SSAKI. Still, when all 21 upregulated gene probes are analyzed using the leave-one-out procedure, the specificity increases to 80%, a level not yet seen for SSAKI biomarkers. Fourth, neither MMP-8 nor elastase-2 has been studied formally in AKI disease pathogenesis, from sepsis or other causes. Since MMP-8 and elastase-2 are neutrophil-derived proteases, it is tempting to speculate that they contribute to neutrophil tissue invasion and end-organ damage. In support of such speculation, our microarray data indicate that septic shock is associated with increased expression of several basement membrane-related genes (for example, laminin-family genes) that contribute to neutrophil diapedesis. Finally, our approach relies on extrapolation of mRNA expression data for the derivation of serum protein-based biomarkers. This can be problematic in that mRNA expression does not always correlate with protein expression. What will be required in the future is exploration into the feasibility of measuring the other candidate biomarkers, such as IFNα-inducible protein 27, which demonstrated a more than twofold mRNA expression level between patients with and without SSAKI.

## Conclusions

In summary, we present a genomic-based methodology that can be used to identify novel candidate biomarkers for SSAKI. Additional validation studies and multicenter studies are required to investigate the potential of SSAKI biomarkers identified by gene array.

## Key messages

• We have used a microarray-based gene expression database to derive a panel of candidate biomarkers for early detection of SSAKI in critically ill children with septic shock.

• The expression patterns of 21 gene probes were able to predict the development of SSAKI with a high degree of sensitivity and specificity.

• The serum protein levels corresponding to two of these gene probes (MMP-8 and neutrophil elastase-2) were able to predict SSAKI with a high degree of sensitivity and a high negative predictive value in both a derivation cohort and a validation cohort.

• The identified candidate genes could provide a foundation to robustly predict SSAKI.

## Abbreviations

AKI: acute kidney injury; CI: confidence interval; IFN: interferon; IL: interleukin; MMP-8: matrix metalloproteinase-8; NGAL: neutrophil gelatinase-associated lipocalin; PICU: pediatric intensive care unit; RIFLE: Risk: Injury: Failure: Loss: End-Stage kidney injury; ROC: receiver operating characteristic; SSAKI: septic-shock-associated acute kidney injury.

## Competing interests

The authors declare that they have no competing interests.

## Authors' contributions

RKB assisted with data analysis and wrote the manuscript. SWS assisted with data analysis and database mining. NZC, GLA, NJT, RJF, NA, KM, PAC, RL, TPS, and MTB contributed patient samples and clinical data to the database. They also reviewed the manuscript prior to submission. DSW, PD, and SLG assisted in the initial study design, assisted with the data analysis, and edited the manuscript. HRW conceived the study, performed the majority of the analyses, and assisted with the writing of the manuscript. All authors read and approved the final manuscript.

## Supplementary Material

Additional file 1 **Gene probes differentially regulated between patients with and without acute kidney injury**. Additional File 1 is a table listing the gene probes identified from mRNA of patient whole blood samples that are differentially regulated (up or down) in patients with acute kidney injury versus those without kidney injury. Click here for file
