# Supplementary material for: Identification of candidate serum biomarkers for severe septic shock-associated kidney injury via microarray
Source: Crit Care. 2011 Nov 18;15(6):R273. doi: 10.1186/cc10554 (PMC3388679; doi:10.1186/cc10554)
Supplement: Additional file 1 — Gene probes differentially regulated between patients with and without acute kidney injury. Additional File 1 is a table listing the gene probes identified from mRNA of patient whole blood samples that are differentially regulated (up or down) in patients with acute kidney injury versus those without kidney injury. [file cc10554-S1.DOC]

**Supplemental Table: One hundred gene probes differentially regulated between patients with and without acute kidney injury.**

| **Affymetrix ID** | **Fold Change*** | **Gene Symbol** | **GenBank ID** | **Description** |
| --- | --- | --- | --- | --- |
| 211990_at | 0.561 | HLA-DPA1 | M27487 | MHC, class II, DP alpha 1 |
| 242772_x_at | 0.57 |  | H48551 |  |
| 244425_at | 0.57 |  | AW573027 |  |
| 236395_at | 0.575 |  | AI523245 |  |
| 201506_at | 0.577 | TGFBI | NM_000358 | transforming growth factor, beta-induced, 68kDa |
| 212998_x_at | 0.578 | HLA-DRB2 | AI583173 | MHC, class II, DR beta 5 |
| 235461_at | 0.586 | TET2 | BG250414 | tet oncogene family member 2 |
| 39318_at | 0.588 | TCL1A | X82240 | T-cell leukemia/lymphoma 1A |
| 1560102_at | 0.593 |  | AL832685 |  |
| 205992_s_at | 0.595 | IL15 | NM_000585 | interleukin 15 |
| 240843_at | 0.595 |  | N53696 |  |
| 244845_at | 0.601 |  | BF725383 |  |
| 213348_at | 0.603 | CDKN1C | N33167 | cyclin-dependent kinase inhibitor 1C (p57, Kip2) |
| 1568934_at | 0.603 | CX3CR1 | AI033393 | chemokine (C-X3-C motif) receptor 1 |
| 1562250_at | 0.603 |  | AF289567 |  |
| 208450_at | 0.605 | LGALS2 | NM_006498 | lectin, galactoside-binding, soluble, 2 |
| 213183_s_at | 0.606 | CDKN1C | N95363 | cyclin-dependent kinase inhibitor 1C (p57, Kip2) |
| 205898_at | 0.606 | CX3CR1 | U20350 | chemokine (C-X3-C motif) receptor 1 |
| 235401_s_at | 0.611 | FCRLA | AL560266 | Fc receptor-like A |
| 239102_s_at | 0.614 |  | AW293296 |  |
| 74694_s_at | 0.62 | RABEP2 | AA907940 | similar to RABEP2 protein |
| 230245_s_at | 0.621 | LOC283663 | AI926479 | hypothetical protein LOC283663 |
| 1558371_a_at | 0.621 |  | BQ899060 |  |
| 203290_at | 0.624 | HLA-DQA1 | NM_002122 | MHC, class II, DQ alpha 1 |
| 239387_at | 0.626 |  | AW004885 |  |
| 238142_at | 0.628 |  | AW029203 |  |
| 235982_at | 0.629 | FCRL1 | AA677057 | Fc receptor-like 1 |
| 213537_at | 0.629 | HLA-DPA1 | AI128225 | MHC, class II, DP alpha 1 |
| 231329_at | 0.629 |  | N21631 |  |
| 210895_s_at | 0.63 | CD86 | L25259 | CD86 molecule |
| 207078_at | 0.632 | MED6 | NM_005466 | mediator complex subunit 6 |
| 234260_at | 0.632 |  | AL122039 |  |
| 205101_at | 0.633 | CIITA | NM_000246 | class II, MHC, transactivator |
| 209823_x_at | 0.634 | HLA-DQB1 | M17955 | MHC, class II, DQ beta 1 |
| 209995_s_at | 0.634 | TCL1A | BC003574 | T-cell leukemia/lymphoma 1A |
| 242974_at | 0.635 | CD47 | AA446657 | CD47 molecule |
| 210889_s_at | 0.637 | FCGR2B | M31933 | Fc fragment of IgG, low affinity IIb, receptor (CD32) |
| 236404_at | 0.637 |  | AW197320 |  |
| 235385_at | 0.638 |  | AI935334 |  |
| 209312_x_at | 0.639 | HLA-DRB2 | U65585 | MHC, class II, DR beta 5 |
| 217478_s_at | 0.64 | HLA-DMA | X76775 | MHC, class II, DM alpha |
| 226844_at | 0.646 | MOBKL2B | AI375115 | Mps One Binder kinase activator-like 2B |
| 1558569_at | 0.646 | UNQ6228 | AL832308 | Hypothetical LOC100131541 |
| 206548_at | 0.647 | hCG_1776259 | NM_024880 | hypothetical protein FLJ23556 |
| 1559078_at | 0.648 | BCL11A | BM193618 | B-cell CLL/lymphoma 11A |
| 223204_at | 0.648 | C4orf18 | AF260333 | chromosome 4 open reading frame 18 |
| 228599_at | 0.648 | MS4A1 | AI862674 | membrane-spanning 4-domains, A1 |
| 222891_s_at | 0.649 | BCL11A | AI912275 | B-cell CLL/lymphoma 11A |
| 215193_x_at | 0.649 | HLA-DRB2 | AJ297586 | MHC, class II, DR beta 5 |
| 230983_at | 0.65 | FAM129C | BE646461 | family with sequence similarity 129, member C |
| 244286_at | 0.651 |  | AI017983 |  |
| 211038_s_at | 0.653 | CROCCL1 | BC006312 | ciliary rootlet coiled-coil, rootletin-like 1 |
| 208146_s_at | 0.654 | CPVL | NM_031311 | carboxypeptidase, vitellogenic-like |
| 211991_s_at | 0.656 | HLA-DPA1 | M27487 | MHC, class II, DP alpha 1 |
| 241860_at | 0.656 |  | AI221707 |  |
| 211656_x_at | 0.657 | HLA-DQB1 | M32577 | MHC, class II, DQ beta 1 |
| 201137_s_at | 0.658 | HLA-DPB1 | NM_002121 | MHC, class II, DP beta 1 |
| 221491_x_at | 0.659 | HLA-DRB2 | AA807056 | MHC, class II, DR beta 5 |
| 205559_s_at | 0.659 | PCSK5 | NM_006200 | proprotein convertase subtilisin/kexin type 5 |
| 214765_s_at | 0.66 | NAAA | AK024677 | N-acylethanolamine acid amidase |
| 203485_at | 0.66 | RTN1 | NM_021136 | reticulon 1 |
| 44790_s_at | 0.661 | LOC728970 | AI129310 | Chromosome 13 open reading frame 18 |
| 210982_s_at | 0.662 | HLA-DRA | M60333 | MHC, class II, DR alpha |
| 227135_at | 0.662 | NAAA | AI436803 | N-acylethanolamine acid amidase |
| 208894_at | 0.663 | HLA-DRA | M60334 | MHC, class II, DR alpha |
| 226877_at | 0.664 | RPL32P3 | AL117606 | ribosomal protein L32 pseudogene 3 |
| 220146_at | 0.664 | TLR7 | NM_016562 | toll-like receptor 7 |
| 236495_at | 0.665 |  | AI681868 |  |
| 227227_at | 0.667 |  | AI344332 |  |
| 240861_at | 0.667 |  | BF433219 |  |
| 204670_x_at | 0.67 | HLA-DRB2 | NM_002125 | MHC, class II, DR beta 5 |
| 1556583_a_at | 0.67 | SLC8A1 | H23209 | solute carrier family 8, member 1 |
| 209200_at | 0.671 | MEF2C | AL536517 | myocyte enhancer factor 2C |
| 244172_at | 0.671 |  | AA931562 |  |
| 1569129_s_at | 0.672 | C3orf38 | BC016013 | chromosome 3 open reading frame 38 |
| 224724_at | 0.672 | SULF2 | AL133001 | sulfatase 2 |
| 1557749_at | 0.673 | EHBP1L1 | AK092750 | EH domain binding protein 1-like 1 |
| 233713_at | 0.673 |  | AK022181 |  |
| 227646_at | 0.674 | EBF1 | BG435302 | early B-cell factor 1 |
| 211821_x_at | 1.486 | GYPA | U00178 | glycophorin A |
| 201292_at | 1.492 | TOP2A | AL561834 | topoisomerase (DNA) II alpha 170kDa |
| 207269_at | 1.497 | DEFA4 | NM_001925 | defensin, alpha 4, corticostatin |
| 211372_s_at | 1.505 | IL1R2 | U64094 | interleukin 1 receptor, type II |
| 218542_at | 1.509 | CEP55 | NM_018131 | centrosomal protein 55kDa |
| 207341_at | 1.512 | PRTN3 | NM_002777 | proteinase 3 |
| 219410_at | 1.517 | TMEM45A | NM_018004 | transmembrane protein 45A |
| 205612_at | 1.522 | MMRN1 | NM_007351 | multimerin 1 |
| 206871_at | 1.525 | ELA2 | NM_001972 | elastase 2, neutrophil |
| 205110_s_at | 1.551 | FGF13 | NM_004114 | fibroblast growth factor 13 |
| 220496_at | 1.601 | CLEC1B | NM_016509 | C-type lectin domain family 1, member B |
| 219478_at | 1.601 | WFDC1 | NM_021197 | WAP four-disulfide core domain 1 |
| 211820_x_at | 1.612 | GYPA | U00179 | glycophorin A |
| 222945_x_at | 1.612 | OLAH | AI125696 | oleoyl-ACP hydrolase |
| 233126_s_at | 1.664 | OLAH | AK001844 | oleoyl-ACP hydrolase |
| 231688_at | 1.666 | MMP8 | AW337833 | matrix metallopeptidase 8 |
| 206145_at | 1.688 | RHAG | NM_000324 | Rh-associated glycoprotein |
| 219975_x_at | 1.697 | OLAH | NM_018324 | oleoyl-ACP hydrolase |
| 212768_s_at | 1.711 | OLFM4 | AL390736 | olfactomedin 4 |
| 207329_at | 1.756 | MMP8 | NM_002424 | matrix metallopeptidase 8 |
| 202411_at | 2.088 | IFI27 | NM_005532 | interferon, alpha-inducible protein 27 |

*****Fold change refers to the median value for patients with acute kidney injury relative to the median value for patients without acute kidney injury.
